# Radiology of fibrosis part II: abdominal organs

**DOI:** 10.1186/s12967-024-05346-w

**Published:** 2024-07-02

**Authors:** Sofia Maria Tarchi, Mary Salvatore, Philip Lichtenstein, Thillai Sekar, Kathleen Capaccione, Lyndon Luk, Hiram Shaish, Jasnit Makkar, Elise Desperito, Jay Leb, Benjamin Navot, Jonathan Goldstein, Sherelle Laifer, Volkan Beylergil, Hong Ma, Sachin Jambawalikar, Dwight Aberle, Belinda D’Souza, Stuart Bentley-Hibbert, Monica Pernia Marin

**Affiliations:** 1 Department of Biomedical Sciences, Humanitas University, Milan, Italy; 2 Department of Radiology, Columbia University Irving Medical Center, New York, NY USA

**Keywords:** Fibrosis, Abdominal organs, Imaging

## Abstract

Fibrosis is the aberrant process of connective tissue deposition from abnormal tissue repair in response to sustained tissue injury caused by hypoxia, infection, or physical damage. It can affect almost all organs in the body causing dysfunction and ultimate organ failure. Tissue fibrosis also plays a vital role in carcinogenesis and cancer progression. The early and accurate diagnosis of organ fibrosis along with adequate surveillance are helpful to implement early disease-modifying interventions, important to reduce mortality and improve quality of life. While extensive research has already been carried out on the topic, a thorough understanding of how this relationship reveals itself using modern imaging techniques has yet to be established. This work outlines the ways in which fibrosis shows up in abdominal organs and has listed the most relevant imaging technologies employed for its detection. New imaging technologies and developments are discussed along with their promising applications in the early detection of organ fibrosis.

**Supplementary Information:**

The online version contains supplementary material available at 10.1186/s12967-024-05346-w.

## Background

This is the second instalment of a three-part series regarding the radiology of fibrosis across organs. This installment concerns abdominal organs, in particular, the pancreas, the liver, and the colon. The prior and subsequent parts of this series are respectively titled “Radiology of Fibrosis Part I: Thoracic Organs” and “Radiology of Fibrosis Part III: Urogenital Organs”. By structuring our work in this manner, we hope to have provided the readership with a clear image of a complex issue, paving the way for future betterment of clinical practice.

As discussed in the first third of this work, fibrosis is the aberrant process of connective tissue deposition resulting from complications in tissue repair following injury [1]. It can affect any organ and is responsible for chronic and debilitating structural and functional impairment of the affected tissue [2, 3]. It has been estimated to account for up to 45% of all deaths in the industrialized world [4]. The profound implications of this datum—both in terms of quality of life and health care burden—argue the need for a more comprehensive understanding of wound healing, the chronic inflammation that may be borne of it, and the fibrosis that ensues. The wound healing mechanism is four-fold and comprises the following: hemostasis, inflammation, proliferation, and remodeling [6–14]. Pathological response to tissue damage may determine an undue protraction of this process resulting in chronic inflammation, aberrant fibroblast proliferation, exaggerated collagen deposition, and a sequent imbalance in the alternation between scar formation and remodeling [3, 5]. Today, chronic inflammation-related fibrosis is widely accepted to be a critical instigator of tumor insurgence, believed to be associated with up to 20% of cancers [2]. The evident gravity of such an assertion highlights the need for a more in-depth knowledge of the interconnectedness of wound healing and fibrosis to encourage subsequent research into cancer insurgence and prevention.

While extensive research has already been carried out on the topic, a thorough understanding of how this relationship reveals itself using modern imaging techniques has yet to be established. Considering the far-reaching implications research furtherance in this field may have—starting from more early and accurate diagnosis—and with the aim of exploring and expanding upon all relevant knowledge, in this work, we have attempted to outline the ways in which fibrosis shows up in the pancreas, liver, and intestines; and have described the most relevant imaging technologies employed for its detection.

## Pancreatic fibrosis

### Mechanism of injury

Pancreatic fibrosis is a hallmark of chronic pancreatitis (CP), defined as the irreversible fibrotic destruction of pancreatic architecture and function [15–17]. The disorder occurs due to recurrent bouts of acute pancreatitis, often progressing to chronic epigastric pain [15]. Annual incidence is low, ranging from 5 to 12/100,000 US adults, and treatment options are limited to lifestyle modifications, pain management, and surgery in case of advanced stage disease [15, 18]. CP’s etiology is multifactorial, having been linked to both genetic and environmental risk factors [15, 19]. The disorder’s evolution in time is tripartite, starting with cellular injury, followed by inflammation, and culminating in fibrosis [19]. Cited environmental determinants include alcohol abuse and nicotine addiction [16]. Studies have shown how the metabolic end products of alcohol’s oxidative and nonoxidative pathways, acetaldehyde and fatty acid ethyl esters, in addition to smoking’s metabolite nitrosamine ketone—derived from nicotine enact direct deleterious effects on pancreatic acinar cells leading to their excessive stimulation of pancreatic stellate cell activity [16, 19]. Similarly, pathologic alterations to one’s genetic makeup have been found to determine cellular dysfunction in the form of increased endoplasmic reticulum stress, oxidative stress, and impaired autophagy, as well as through the pathological alteration of the pancreatic ductal cells’ secretion of bicarbonate [19]. Cell injury and death result in inflammation, initiated by NF-κB and perpetuated by innate immune cells, predominantly macrophages [17, 19]. When excessively prolonged, this physiological response to pathological stimuli leads to excess deposition of extracellular matrix (ECM) and tissue remodeling, ultimately resulting in interlobular and intralobular fibrosis, acinar cell loss, distorted architecture, dilated ducts, and loss of function [18, 19]. When 90% of pancreatic activity is compromised, patients present with signs of exocrine and endocrine insufficiency: steatorrhea, malabsorption, fat soluble vitamin deficiencies, and the development of diabetes mellitus type 2 [15, 17].

The diagnosis of pancreatic fibrosis is challenging, relying solely on clinical anamnesis and imaging findings [15, 20]. To date, the most relevant imaging techniques comprise trans-abdominal US, endoscopic US (EUS), endoscopic retrograde cholangiopancreatography (ERCP) (considered the diagnostic gold standard tool for pancreatic ductal investigation), computed tomography (CT), magnetic resonance imaging (MRI), magnetic resonance cholangiopancreatography (MRCP), MRCP with secretin stimulation (S‐MRCP), and Elastography [15, 20, 21].

### Ultrasound (US)

Conventional transabdominal gray-scale B-mode US is often the first radiological assessment performed to evaluate the pancreas given its great availability, low cost, and lack of ionizing radiation [21–23]. While US is seldom useful in early-stage detection of CP, common pancreatic parenchymal findings later in the disease process include increased gland dimensions, altered echogenicity with mixed areas of hyperechogenicity (representing fibrotic tissues and pancreatic calcification) and hypoechogenicity (representing inflammatory tissues), dilatation and irregularity of the pancreatic duct [21, 23, 24]. Use of transabdominal US may be limited by the retroperitoneal location of the gland [22]. Overlying bowel gas shadows often cause partial or complete obscuration [21, 23]. Image quality is heavily dependent on patient body habitus and the radiologist’s skill [21, 23].

US’ limitations relative to patient body build and gaseous abdomen are overcome by endoscopic ultrasound (EUS) [21–23]. EUS is a common diagnostic tool for CP because of its superior spatial resolution, helping to evaluate subtle morphologic changes in the pancreatic parenchymal structure and allowing for early-stage diagnosis of pancreatic fibrosis [15, 20, 21, 23–25]. Indeed, placement of high-frequency transducers in close proximity to the pancreas increases resolution allowing for improved imaging [21, 23]. This technology has been reported to have high sensitivity (81–97%), specificity (60–90%), and diagnostic accuracy [15, 20, 21]. Drawbacks of EUS are its considerable intra- and interobserver variability and considerable false positivity rate given that some findings may occur normally with aging, in smokers and in alcoholics [21, 23]. Furthermore, this modality is invasive and presents a non-negligible risk of postprocedural complications [24].

As in all fibrosis affected tissues, stiffness elevation is a determining characteristic of pancreatic fibrosis and, consequently, could be quantified via the elasticity-based imaging technologies such as USE [21, 22, 26, 27]. USE is a noninvasive and real-time US based elastography technique which helps to quantitatively measure the stiffness of a tissue to assess fibrosis of the pancreas in CP [21, 22, 25, 26, 28]. USE can be classified into two categories: strain elastography (SE) and shear-wave elastography (SWE) [20, 21, 25]. In USE-SE, the strain created by compression of the target tissue with the US probe is measured: a larger strain indicates softer tissue [20, 25]. In USE-SWE, instead, an acoustic radiation force is sent to a focal point within the tissue and a shear wave is generated [20, 21, 25]. Consequently, the shear wave velocity is calculated: if the tissue is hard, the shear wave propagates faster [25]. Both SWE and SE yield elastograms, which are colored elasticity maps superimposed onto tissue images, although USE-SWE is the more precise modality for diagnosing CP because it can provide absolute values of pancreatic hardness [21, 25]. USE is currently considered to be the most sensitive—71% to 91%—and specific—86% to 100%—modality for diagnosing CP [26, 27]. Even so, it presents inadequate standardization in mode of execution, evaluation, and choice of terminology inducing discord among professionals [20, 26]. Moreover, it has also been found to have limited reliability in patients who smoke, abuse alcohol, are obese, and in the elderly [20, 27].

### Computed tomography (CT)

Contrast-enhanced CT (CE-CT) is the preferred imaging technique in case of suspected chronic pancreatitis given its non-invasivity and ubiquity, providing highly resolute images within seconds, with high sensitivity and specificity [15, 20, 22, 24, 25, 29]. While its detection of early structural CP related fibrotic changes is not reliable, this technology has been reported to have high sensitivity (60‐95%), specificity (85–91%), and diagnostic accuracy later in the disease [15, 20, 21, 23, 25]. Multiphase protocol is now commonly used in the assessment of pancreas [21]. It includes a precontrast unenhanced sequence to identify calcifications, a pancreatic or late-arterial phase to assess arterial complications, and a portal venous phase to evaluate the parenchyma, pancreatic duct, focal lesions, pancreatic masses or complications from pancreatitis [21, 25, 30]. This method allows for the detection of morphological alterations, such as pancreatic ductal calcifications (pathognomonic findings of chronic pancreatitis), dilation of the main pancreatic duct and side branches secondary to traction from periductal fibrosis, altered size and shape of the gland, pseudocysts, pseudoaneurysms, vascular thrombosis, necrosis, and parenchymal atrophy [15, 22–25, 30, 31]. The main drawback to the application of CE-CT is the radiation exposure to which patients are subject, especially since this chronic disease state often calls for serial monitoring [20, 22]. When CT results are inconclusive, magnetic resonance imaging (MRI), magnetic resonance cholangiopancreatography (MRCP), EUS, and endoscopic retrograde cholangiopancreatography (ERCP) may be used [15].

#### MRI

MRI is an alternative imaging modality for those in whom CT or ERCP is contraindicated or not tolerated [32]. Indeed, it is a non-invasive method for the early recognition of pancreatic fibrosis having excellent soft-tissue contrast, with high sensitivity (78%) and specificity (96–100%) [15, 20, 21, 24, 32]. MRI’s main drawback consists of its high cost [20]. Due to the high content of proteinaceous enzymes, the normal pancreas typically appears diffusely hyperintense on T1-weighted images [21, 22]. In CP, chronic inflammation and fibrotic replacement of parenchyma diminish the proteinaceous fluid content of the pancreas resulting in heterogenous hypointense areas on T1-weighted imaging and heterogenous and mildly hyperintense on T2-weighted images with diminished and heterogenous parenchymal enhancement after administration of intravenous gadolinium agents [22, 23, 25, 30, 32–34].

**Fig. 1:**
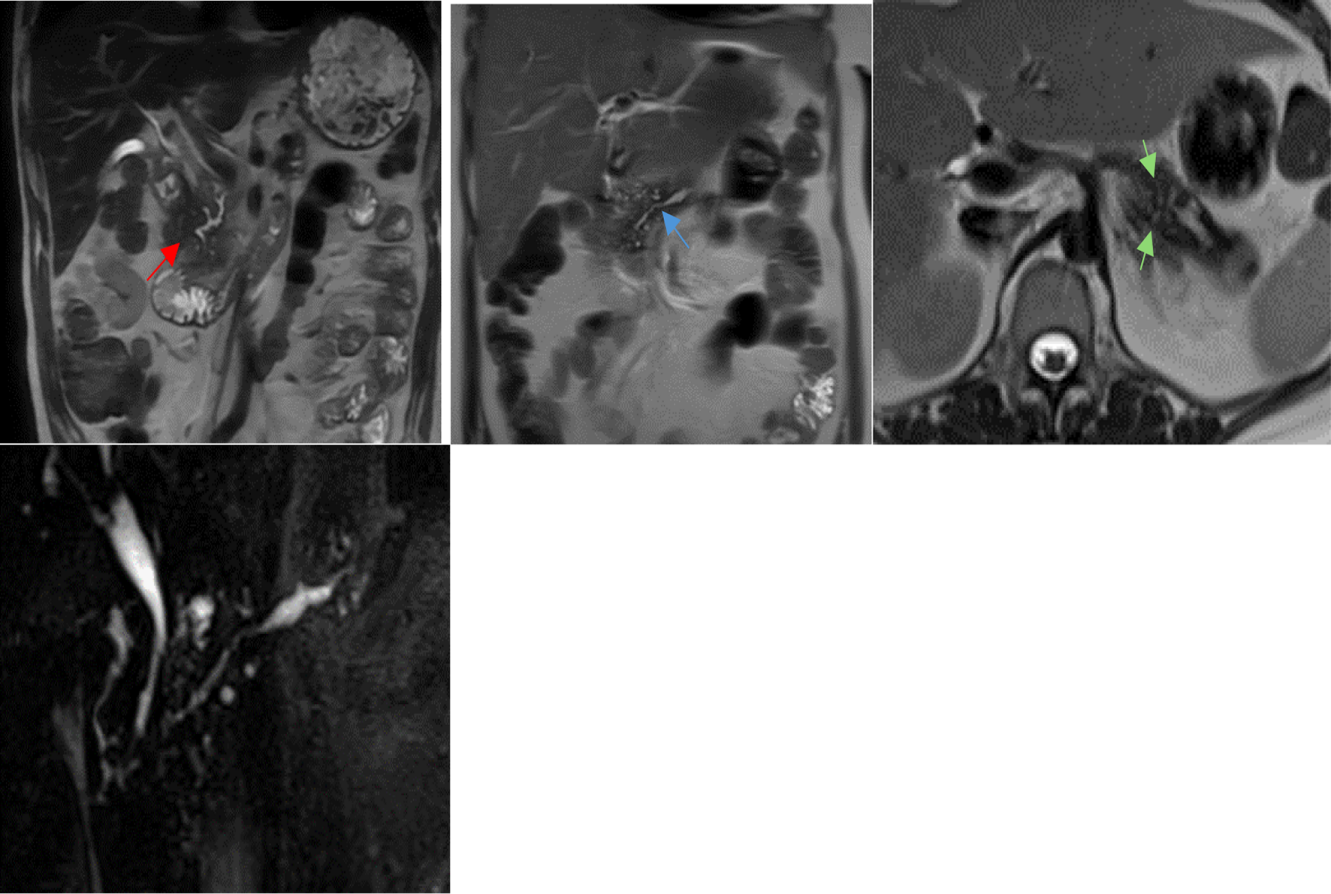
MRI (coronal T2 and axial) and MRCP from two patients with crhonic pancreatitis, showing T2 hypointense pancreatic signal (red arrow), tortuosity of the main pancreatic duct (blue arrow), and numerous prominent side branches (green arrows)

MRCP is the most effective, safe, noninvasive MR imaging technique for the evaluation of the pancreatic parenchyma, main pancreatic, and common bile ducts [15, 21–23, 25]. It presents with high sensitivity (78%), specificity (96%), and diagnostic accuracy [21]. It only makes use of nonionizing radiation and for this reason it is increasingly used in the diagnosis of CP [15, 23, 25, 30]. MRCP is the preferred alternative to ERCP in patients for whom this imaging modality has failed or is not tolerated [21, 32]. Even so, the typical calcifications in chronic pancreatitis are not visualized as effectively as on CT and the evaluation of side branches is less sensitive than in ERCP [15, 30]. Addition of secretin enhancement to MRCP (S‐MRCP) can improve morphological and functional assessment of abnormalities of the main pancreatic duct and its side branches, which may not be seen on routine MRCP [21–23, 25, 30, 32]. Secretin is a polypeptide amino acid which is normally secreted by the S cells of the duodenal mucosa and can be synthetically purified [21, 22]. Its physiological effects include stimulation of the pancreas to secrete fluid and bicarbonate from acinar cells into the duodenum, thus increasing the absolute volume of intraductal free water and filling the collapsed branches [21–23, 25]. Additionally, secretin increases the tone of the sphincter of Oddi, thus hindering the release of this accumulated fluid through the papilla of Vater, and making it easier to distinguish the main pancreatic duct and its branches [23, 25]. In S‐MRCP, pre‐secretin images are obtained before the polypeptide is injected intravenously after which a series of T2‐weighted images are acquired [21, 23]. In cases of CP, a lack of ductal compliance results in dilated side branches [21]. By injecting intravenous secretin, MRI can also diagnose chronic pancreatitis by evaluating exocrine secretion response [24]. Even so, S-MRCP lacks proper analysis of parenchyma, thus limiting its use [20]. Axial and coronal T2 weighted MRI and MRCP images of a liver affected by CP are reported in Fig. 1. Note how hypointense the pancreatic signal is on T2, the tortuosity of the main pancreatic duct, and its numerous prominent side branches.

#### Other

ERCP is a combined endoscopic and fluoroscopic procedure mainly used in the diagnosis of early CP with high sensitivity (71–95%), specificity (89–100%), and diagnostic accuracy [15, 21, 25, 29]. For these reasons, it is currently considered the diagnostic gold standard tool for pancreatic ductal investigation. It has great spatial resolution and the ability to depict side branch abnormalities, characteristic of early disease [25, 32]. In ERCP, an endoscope is advanced into the second part of the duodenum, thus allowing other tools to be passed into the biliary and pancreatic ducts via the major duodenal papilla [29]. Contrast material injected into these ducts, allows radiologic visualization of pancreatic duct abnormalities—ductal dilation, stricture, abnormal side branching, communicating pseudocyst, pancreatic duct stone, and pancreatic duct leakage—and therapeutic intervention—dilation for pancreatic duct stenosis, stone extraction, and stenting of the pancreatic duct [25, 29, 32]. This technique is, however, the most invasive of the diagnostic modalities for CP, only allows for visualization of duct anatomy and not that of pancreatic parenchyma, and is associated with a high risk of complications [15, 23–25]. The possibility for adverse events directly attributed to ERCP is as high as 6.8% and include post-ERCP pancreatitis, infections, gastrointestinal bleeding, duodenal and biliary perforations [25, 29]. For all these reasons, ERCP should be performed only when all other tests are inconclusive [15, 25].

#### Future directions

Promising future techniques, benefits, and drawbacks of each imaging technique discussed above are summarized in Table 1. Among the proposed alternatives, the authors of this review believe MRCP (Fig. 1) and USE to be the most promising. Indeed, USE is currently considered to be the most sensitive—71% to 91%—and specific—86% to 100%—modality for diagnosing CP provided that standardization in mode of execution, evaluation, and choice of terminology be enacted [20, 26, 27]. USE is a noninvasive and real-time US based elastography technique which helps to quantitatively measure the stiffness of a tissue, a determining characteristic of pancreatic fibrosis [15, 21, 22, 25, 26]. Both USE sub modalities—SWE and SE—yield elastograms, which are colored elasticity maps superimposed onto tissue images to help locate fibrotic areas [20, 21, 25]. Instead, MRCP presents with high sensitivity, specificity, and diagnostic accuracy [21]. It only makes use of nonionizing radiation and for this reason it is increasingly used in the diagnosis of CP [15, 23, 25, 30]. Addition of secretin enhancement to MRCP (S‐MRCP) can improve morphological and functional assessment of abnormalities of the main pancreatic duct and its side branches, which may not be seen on routine MRCP [21–23, 25, 30, 32].

**Table 1:** Pancreatic fibrosis imaging—Pros/Cons with respects to the gold standard

Pancreatic fibrosis imaging		
US	PROs	Readily available, low cost, lack of ionizing radiation
	CONs	Limited by pancreases location and patient habitus, operator dependent
EUS	PROs	High spatial resolution, early-stage diagnostic, high sensitivity/specificity/accuracy
	CONs	High inter-observer variability, high false positive rate, invasive, high rate of complications
USE-SE/SWE^b^	PROs	Quantitative, high sensitivity/specificity
	CONs	Operator dependent, low reliability in certain patient subgroups
CE-CT^a^	PROs	Readily available, high spatial resolution, fast, high sensitivity/specificity/accuracy
	CONs	Late diagnosis, use of contrast agents, use of ionizing radiation
MRI	PROs	Early diagnosis, high soft tissue contrast, high sensitivity/specificity, no ionizing radiation
	CONs	High cost, use of contrast agents, time consuming
MRCP/S-MRCP^b^	PROs	High sensitivity/specificity/accuracy, no ionizing radiation
	CONs	Does not allow for proper visualization of calcification/side branches/parenchyma, Invasive
ERCP	PROs	High sensitivity/specificity/accuracy, early diagnosis
	CONs	Invasive, high rate of complications, does not allow for proper visualization of parenchyma

^a^Gold standard

^b^Promising future techniques

## Liver fibrosis

### Mechanism of injury

Chronic liver disease (CLD) is characterized by progressive deterioration of liver function due to persistent inflammatory response, parenchymal injury and regeneration leading to abnormal wound healing and, ultimately, liver failure [35–37]. CLD etiology is varied and determines the patterns of liver fibrosis [35, 37]. Among the most notable causes are toxins, excessive alcohol consumption, viral and autoimmune hepatitis, as well as genetic and metabolic disorders [35, 37]. Since the end of the last century, the incidence of CLD has undergone a 62.03% increase worldwide. In line with this datum is the CDC’s estimates of the number of American adults affected by CLD being 4.5 million, about 1.8% of the population, making it of great clinical relevance [36, 38]. The aberrant accumulation of ECM that follows CLD onset is triggered by injured hepatic stellate cells (HSC) and inflammatory cells’ paracrine stimulation which induces rapid gene conversion of quiescent HSCs into proliferative myofibroblasts [35, 37, 38]. This fibrotic response is perpetuated by cellular events that amplify the activated phenotype through enhanced growth factor expression leading to fibrous scar formation [39]. Only the withdrawal of injury-causing stimuli can promote the spontaneous resolution of hepatic fibrosis, otherwise, CLD can progress into cirrhosis, a pre-malignant condition that may ultimately lead to hepatocellular carcinoma [35, 37, 39]. Through senescence and apoptosis, the levels of cytokines and myofibroblasts lowers, triggering, in turn, the start of fibrotic regression by decreasing the levels of tissue inhibitors of metalloproteinase (TIMPs) and by increasing the levels of matrix metalloproteinases (MMPs) [35, 39]. In so doing, TIMPs are kept from inactivating collagenases and exercising their antiapoptotic influence on stellate cells, while MMPs’ type I collagenase activity is encouraged to effectively cleave collagen and other matrix components [35, 39]. When withdrawal of injury-causing stimuli is not possible, persistent fibrosis leads to remodeling of the hepatic parenchyma and development of a shrunken nodular contour, detectable via imaging and pathology [35, 36].

Traditionally ultrasound—one of the most common and affordable techniques—and CT—more precise than the previous—have been used to assess the presence of fibrosis in the liver, focusing on gross morphological changes of the organ’s architecture [38, 40]. Unfortunately, these methods do not allow for detection of less advanced stages of fibrosis [40]. A need which is, instead, met by transient elastography (TE) and magnetic resonance elastogragphy (MRE), the most widely used novel hepatic fibrosis assessment methods in Europe [38, 40]. They are rapid, noninvasive, and reproducible [40]. TE and MRE measure the velocity of a mild amplitude and low frequency (50 Hz) elastic shear wave travelling through the liver [38, 40]. The wave speed is measured and used to approximately quantify tissue stiffness: the faster the wave, the stiffer the tissue [38]. It has been estimated that these novel imaging techniques eliminate the need for liver biopsy in up to 70% of patients as well as allowing for early detection of reversible liver fibrosis, thus greatly reducing morbidity and mortality [40–42]. It is important to note, however, that increased liver stiffness is not always a satisfactory proxy for fibrosis [40].

When withdrawal of injury-causing stimuli is not possible, persistent fibrosis leads to remodeling of the hepatic parenchyma and development of a shrunken nodular contour, detectable via imaging and pathology [35, 36]. Traditionally US, MRI, and CT have been used to non-invasively diagnose and stage hepatic fibrosis, focusing on gross morphological changes of the organ’s architecture [40, 43]. However, it has been found that these methods do not allow for reliable detection of less advanced stages of fibrosis [40]. A need which is, instead, met by US and MR elastography [38, 40, 43]. Other diagnostic methods include diffusion weighted imaging, MRI with hepatobiliary contrast agents, MR and CT perfusion, dual energy CT, contrast-enhanced US (CEUS), image texture analysis, and Magnetization transfer imaging [43–46]. It has been estimated that these novel imaging techniques eliminate the need for liver biopsy in up to 70% of patients as well as allowing for early detection of reversible liver fibrosis, thus greatly reducing morbidity and mortality [40–42].

#### US

In patients with suspected CLD, liver US is the first modality employed, because it is widely available, ionizing radiation-free, and less expensive than its alternatives [38, 45, 47]. US findings that suggest fibrotic disease include coarse surface nodularity and increased parenchymal echogenicity [45, 48]. In the early stages of CLD, however, these findings present with low sensitivity and specificity [45]. Indeed, other conditions, such as steatosis may also lead to brighter image acquisition, resulting in a potential for confusion [48]. Finally, obesity reduces the accuracy of US due to increased attenuation of signal by subcutaneous fat [48].

In time, USE has become the leading US-based alternative to basic US for the detection and staging of liver fibrosis [40, 47, 50, 53, 56]. The impulse’s sheer wave velocity and resultant tissue displacement is dependent on tissue elasticity which has been found to decrease with increasing fibrosis [48–50]. Thus, elastography techniques quantify increased tissue stiffness as proxy for fibrosis, even in early stages [47, 49, 50, 57]. USE is currently the most widely used noninvasive means of quantifying hepatic fibrosis [40, 51]. It may be subdivided into vibration-controlled TE (VCTE), point sheer wave elastography (pSWE), and two-dimensional SWE (2D-SWE) [41, 49, 50].VCTE is a one-dimensional technique that uses a mechanical driver to generate a low-frequency sheer wave whose velocity across the liver parenchyma is measured using sonographic Doppler [38, 40, 49, 50]. Intraobserver agreement for VCTE is excellent having high repeatability and reproducibility and requiring little dedicated training time [49, 50]. It has demonstrated high accuracy for advanced fibrosis; however, diagnostic performance is more modest in case of lesser degrees of fibrosis [49, 50]. Furthermore, this technology is subject to several technical and patient-related limitations. Indeed, technical failure rate increase in the presence of confounders such as acute inflammation, narrow intercostal space, ascites, increased steatosis, and obesity [49, 50].

In pSWE, a high frequency sonographic impulse generates a single push pulse into a focal point in the liver [49, 50]. This shear wave’s velocity is measured via conventional pulse echo US [38, 50]. Interpretation of pSWE is aided by incorporation into a standard B-mode US device which allows the operator to visualize the liver tissue [50]. Instead, in 2D-SWE, a high frequency sonographic impulse generates shear waves at multiple points, producing a cone-shaped shear wave front which is monitored in real-time at multiple spatial and temporal points using 2D US waves and is ultimately depicted as a colorized elasticity map known as an elastogram [49, 50]. In general, SWE presents with good interobserver variability (greater in 2D-SWE), as well as excellent repeatability and reproducibility having low scan failure rate following an initial learning curve [49, 50]. Despite recent evidence showing high diagnostic accuracy for diagnosing advanced fibrosis stages, they do not perform as well in case of lower liver fibrosis [50]. Both are susceptible to motion and, thus, require breath holding [50].

#### CT

Conventional no-contrast-medium CT scans have been found to be useful in assessing morphological liver changes—stage, extent, and distribution of fibrosis—with positive correlation between histological and CT findings depending on the homogeneity of the fibrosis distribution [45, 51, 52]. Radiographic density on CT full-liver analysis allows for more highly accurate and precise diagnosis of fibrosis than in US [38, 48, 51, 52]. However, the use of ionizing radiation confers increased patient risk to this technique, making it less suited for repeated measurements [48]. Similar to US, CT is less sensitive for less advanced stages of liver fibrosis [45, 51].

#### MRI

**Fig. 2:**
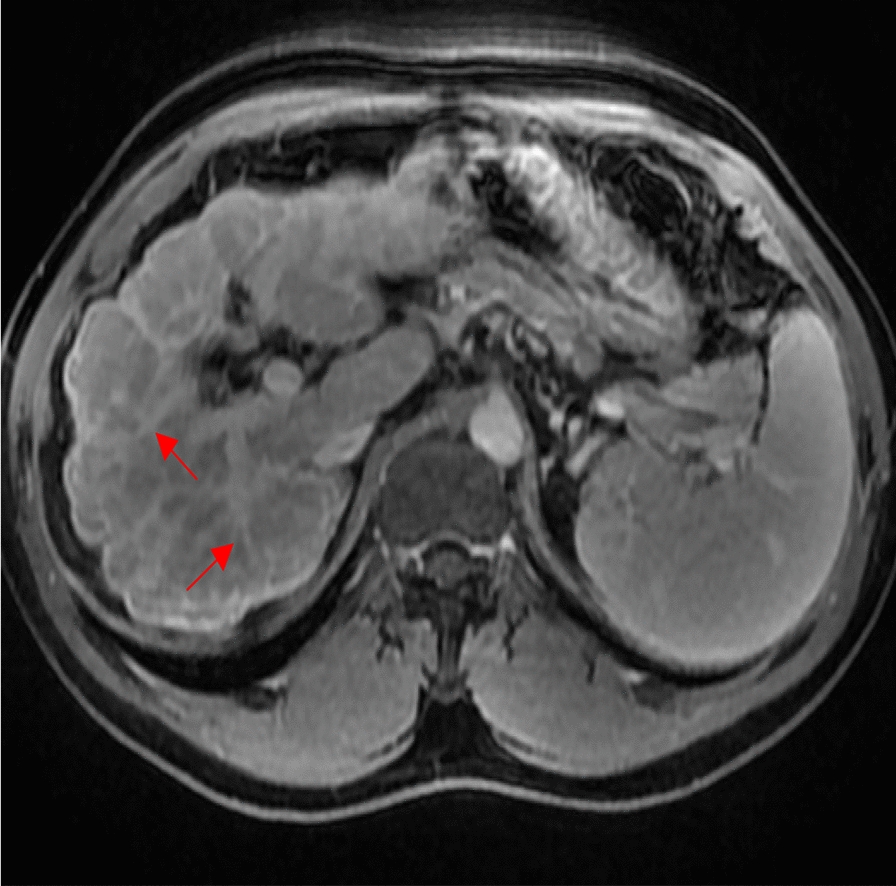
Axial T1 Weighted post contrast sequence with fat suppression demonstrates hepatic fibrotic bands

This same shortcoming is presented by conventional MR imaging as the presence of hepatic fibrosis generally causes little anatomic change in the liver until late in the disease [45, 51, 53]. In attempts to more reliably stage hepatic fibrosis, mapping of T1 relaxation time, which has been found to be positively correlated to increased levels of ECM, inflammation, and fibrosis, may be adopted [48]. Indeed, by comparing histological data to hepatic T1 mapping, Pavlides et al. were able to determine optimal T1 cut-off values and create a liver inflammation and fibrosis staging score with which to classify hepatic fibrosis [48, 54]. Further research is needed to validate this scoring system [48]. In Fig. 2, hepatic bands of fibrosis can be seen on a post contrast T1 weighted axial MRI image with fat suppression.

Along with morphological T1 mapping, several alternative MRI-based imaging techniques have been developed [55]. These include texture analysis MRI, spin–lattice relaxation time mapping in the rotating frame (T1q), diffusion-weighted imaging, perfusion MRI, and the use of hepatobiliary contrast agents, for all of which, studies have demonstrated a clear correlation to increased liver fibrosis [53, 55].

Among these alternative MRI-based imaging techniques, MRE has emerged as a leading non-invasive, objective, and quantitative alternative method for the detection and staging of liver fibrosis [40, 47, 50, 53, 56].

**Fig. 3:**
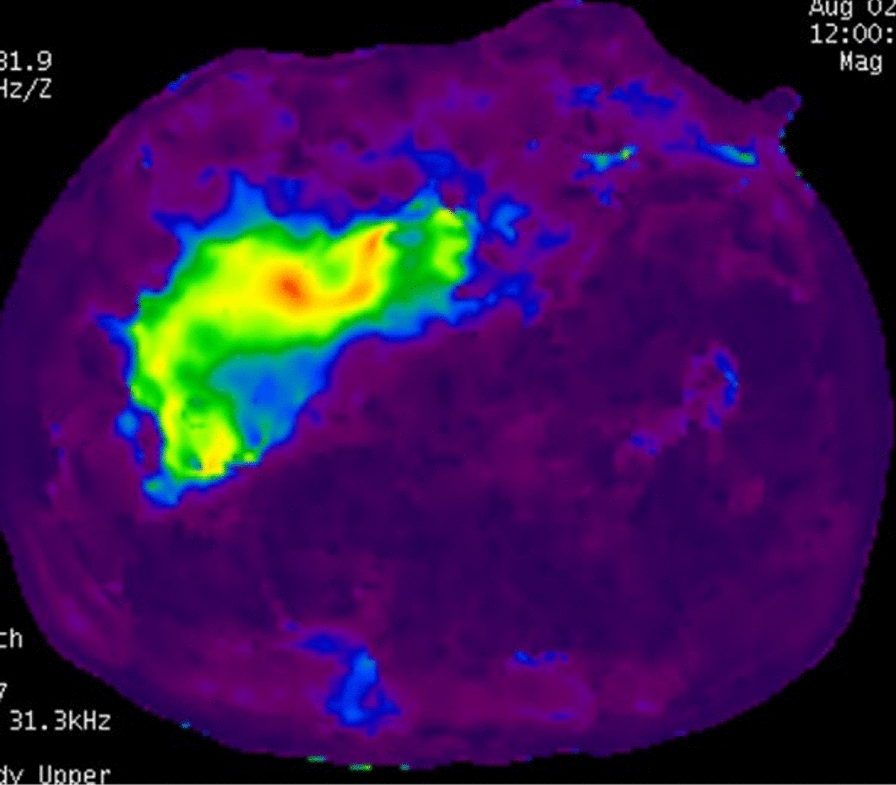
Liver MR elastography examination. Red and yellow areas represent highest liver stiffness measurements within the right hepatic lobe consistent with fibrosis

**Fig. 4:**
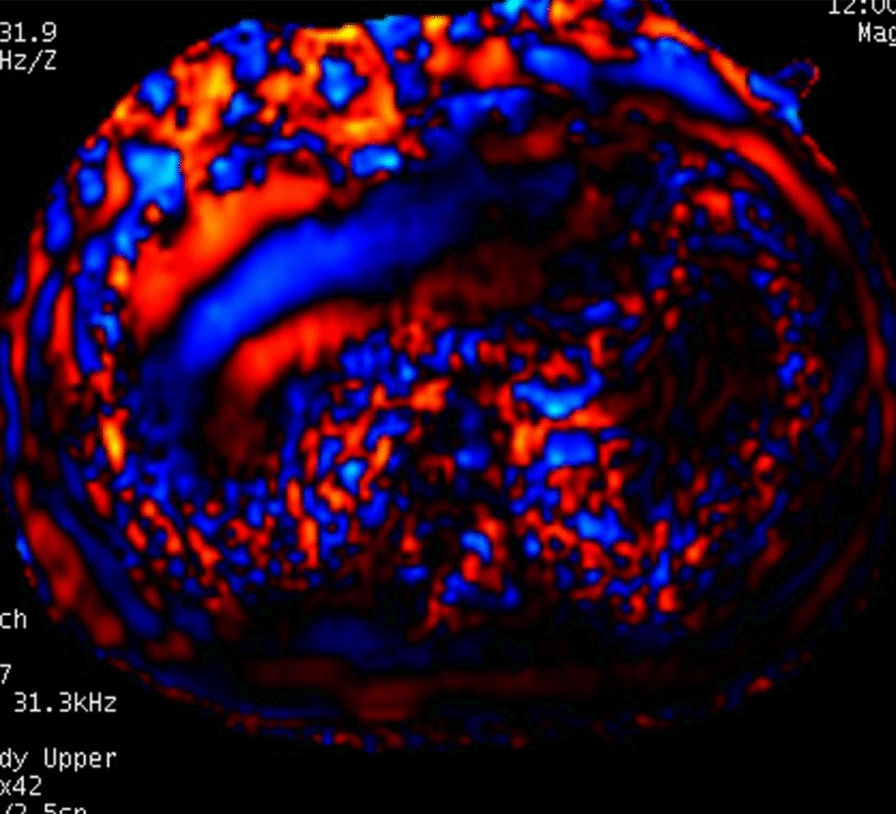
Shear wave image demonstrates waves that are thicker than normal. This is because they move more quickly through the stiffer, fibrotic liver parenchyma

It is considered the most accurate noninvasive imaging technique for detecting and staging liver fibrosis [40, 51, 53]. It may be subclassified into two-dimensional MRE (2D-MRE), currently the gold standard for hepatic fibrosis detection, and three-dimensional MRE (3D-MRE) [50]. In 2D-MRE, an external acoustic driver system generates low-amplitude vibrations [38, 40, 47, 50, 53]. Resultant shear waves propagate in a largely transverse manner, allowing analysis of wave motion by MR sequences to be carried out only in a single 2D plane [38, 48, 50, 53]. The acquired wave images are post-processed to generate a color-scaled representation of tissue stiffness known as an elastogram [50, 53]. By examining a wider portion of liver in comparison to that examined by USE, MRE appears more accurate and is less prone to sampling error, ultimately producing more representative maps of liver stiffness [47–49]. Technical failure is rare (≤ 5%) and is mostly determined by the presence of excess iron in liver parenchyma [49, 50, 53]. Indeed, iron causes T2 shortening and signal loss, which diminishes the visibility of shear waves on phase contrast images [50]. Furthermore, being a motion-sensitive technique, a fraction of the failure rate is due to motion artifacts [50]. 2D-MRE benefits from robust repeatability and reproducibility between radiologists, it calls for an extremely short acquisition time (1–2 min) and can be included in any standard MRI exam of the liver [47, 49, 50, 53]. Even so, it is not yet recommended in routine clinical practice given its cost, limited availability, and a minority of patients’ inability to tolerate MR exams due to claustrophobia, inability to fit into the MR scanner bore, or having been implanted with MR-incompatible devices [47, 50]. Instead, 3D-MRE is an emerging imaging modality, mainly used in research settings, which carries out analysis of wave motion in a 3D volume rather than in a single 2D plane [50]. Although they have been demonstrated to be more accurate in predicting advanced fibrosis than 2D-MRE, further validation is required prior to recommending it for routine clinical use [49, 50]. Finally, the diagnostic performances of elastography techniques are set to be maximized by artificial intelligence in the near future [47]. In fact, this technology promises to achieve high diagnostic performance and high accuracy for the prediction of fibrosis stages, largely outperforming radiologists [47]. In Fig. 3, tissue displacement subsequent to harmonic shear wave induction is depicted. Areas in which wavelengths are longer correspond to stiffer areas. This wave data is then converted into a shear stiffness elastogram In Fig. 4, an example of such an elastogram in which areas of highest liver stiffness measurements appear red and yellow is provided.

#### Future directions

Promising future techniques, benefits, and drawbacks of each imaging technique discussed above are summarized in Table 2. Among the proposed alternatives, the authors of this review believe AI supplemented 3D-MRE to be the most promising. Indeed, preliminary data has shown 3D-MRE – an emerging imaging modality which carries out analysis of wave motion in a 3D volume rather than in a single 2D plane – to be more accurate in predicting advanced fibrosis than 2D-MRE [49, 50]. Furthermore, the diagnostic performance of such elastography techniques is set to be maximized by AI in the near future [47]. The pairing of these technologies promises to achieve high diagnostic performance and high accuracy for the prediction of fibrosis stages, largely outperforming human radiologists [47].

**Table 2:** Liver fibrosis imaging—Pros/Cons with respects to the gold standard

Liver fibrosis imaging		
US	PROs	Readily available, low cost, lack of ionizing radiation
	CONs	Low sensitivity/specificity, dependent on patient habitus, operator dependent
USE-VCTE/SWE^a^	PROs	Quantitative, lower operator dependency, high accuracy in advanced stages
	CONs	Low accuracy in early stages, decreased performance in case of acute inflammation/narrow intercostal space/ascites/steatosis/obesity, motion sensitive
CE-CT	PROs	High accuracy, readily available, fast
	CONs	Use of contrast agents, use of ionizing radiation, low sensitivity in early stages
MRI	PROs	Quantitative, no ionizing radiation
	CONs	Low sensitivity in early stages, high cost, time consuming
2D/3D-MRE^a,b^	PROs	High accuracy, low sampling error due to larger field of view, high repeatability, fast
	CONs	Motion dependent, high cost, not readily available, not suitable for patients with claustrophobia/MR incompatible implants/obesity

^a^Gold standard

^b^Promising future techniques

## Intestinal fibrosis

### Mechanism of injury

Intestinal fibrosis can develop from several conditions, including chronic ischemic enteritis, radiation enteritis, cystic fibrosis and, most importantly, inflammatory bowel diseases (IBD). IBD, comprising Crohn’s disease (CD) and ulcerative colitis (UC), consists of an exaggerated, recurrent inflammatory response to bowel injury leading to disorganized ECM deposition [58–61]. Ultimately, CD and UC’s protracted course of relapse and remission leads to bowel damage, weakened barrier function, and disability [58, 61–63]. Its prevalence, while increasing worldwide, was estimated to be more than 3 million in the USA and Europe by a 2017 Global Burden of Disease Study [61, 62, 64]. Prevalence is greatest among industrialized nations and metropolitan areas [61]. However, low-risk regions have experienced a marked surge in IBD rates, in concordance with their development and adoption of traditionally “western” lifestyles, thus implicating environmental factors in CD and UC pahtophysiology [61]. The most studied of these influences are cigarette smoking, associated with a two-fold increase in CD risk, and dietary imbalance, in particular, a reduction in dietary fiber and an increase in saturated fat intake leading to dysbiosis [61]. Additionally, more that 200 allelic mutations have been found to be positively associated with IBD incidence [61, 63]. Even so, only 13% of the disease’s transmission can be explained this way, emphasizing once more environmental determinants’ role in CD and UC development [60, 61, 63]. Clinically, CD manifests with abdominal pain, chronic diarrhea, weight loss, and typically segmental and transmural gastrointestinal (GI) inflammation [58, 61, 62]. The excess secretion of ECM in intestinal fibrosis is made possible by intestinal mesenchymal cell expansion [59, 62]. Primarily that of fibroblasts, myofibroblasts, and smooth muscle cells [62]. Immune cells contribute to these fibrotic processes by secreting IL-17A and IL-13 cytokines [62]. These augment mesenchymal cell activation, thus promoting scar formation through positive feedback loops [62]. In particular, IL-17A is found to be upregulated in the mucosa and lamina propria of CD patients [62]. Myofibroblasts upregulate their receptors for these proteins, resulting in their reduced migratory ability as well as increased ECM production [62]. Similarly, IL-13, Th-2 cells’ most potent fibrogenic mediator, facilitates ECM deposition through increased TGF-β1 secretion [62]. Furthermore, a sharp downregulation of matrix metalloproteinases (MMPs), enzymes meant to degrade deposited ECM, and overexpression of TIMPs, MMP inhibitors, further favors uncontrolled ECM synthetization [58]. Abnormal wall thickening and contraction ultimately lead to tissue distortion and increased stiffness [60, 62]. This may take place at any time during IBD progression and occurs at equal rate in all segments of the gut [60, 62]. The most common clinical sequelae of intestinal fibrosis, occurring in more than half of all CD patients within 10 years of diagnosis, are strictures, abscesses, and fistulae, predominantly in the terminal ileum and the ileocolonic region [58, 61, 62]. In turn, these cause bowel obstruction, requiring anti-inflammatory, endoscopic, and/or surgical relief [62]. Secondary to intestinal obstruction, patients experience muscularis propria hypertrophy, which results in peristaltic abnormalities [60]. CD diagnosis relies on a combination of clinical, imaging, histological, blood, and stool findings [65, 66]. Choosing which of these strategies to put in place depends on the patient's age, pregnancy status, general health, and availability [67].

The current gold standard imaging technique is endoscopic evaluation via ileo-colonoscopy [65, 66]. This procedure is widely available and well tolerated among patients despite its invasiveness [65, 68]. It allows for direct inspection of the GI lumen, facilitating physicians in identifying common lesions and overseeing treatment progression [67]. Endoscopically, CD may manifest as mucosal nodularity, swelling, ulceration, and narrowing [66]. However, while the vast majority of those affected by IBD will have colonoscopically detectable sequalae, this technique cannot ensure satisfactory imaging of extraluminal and intramural inflammation, the small intestine—the most commonly affected segment of the GI tract—or the intestine beyond a stricture [65, 66, 68]. Moreover, interobserver variability, the risk of bowel perforation, the need for bowel preparation, and the occasional need for anesthesia comprise some of endoscopy’s major limitations [65]. For all these reasons, CD complications are often best identified via small bowel imaging techniques, the most popular of which are US, CT, and MRI [66, 68]. These allow for the identification and examination of pathology not accessible through ileo-colonoscopy [67]. Other promising technologies are transabdominal USE, CEUS, DWI, and magnetization transfer MRI (MT-MRI). US is recommended as a first-line test for the assessment of inflammatory lesions and long-term follow-up of CD given its non-invasivity, lack of ionizing radiation, increased availability, relatively low cost, and real-time capabilities [65, 68, 70]. It has proven to be as sensitive and specific as MR, CT, and endoscopy for detecting IBD [65]. Even so, it is highly operator-dependent, limited by disease location and patient body build, with limited reproducibility and generalization [68].

#### US

Transabdominal USE is a promising real-time bowel imaging technique. It has been designed to indirectly assess bowel fibrosis in CD through the direct evaluation of intestinal wall stiffness. Its main drawback is given by its operator dependent nature as well as its poor performance on deep bowel loops [69–71]. There are two main elastographic subtypes: US-SE and US-SWE [70]. In US-SE, an external force applied to a fixed area of the tissue under investigation evokes a strain, the measurement of which allows for the estimation of tissue stiffness [68, 70–72]. This noninvasive assessment of tissue mechanical properties is useful seeing as strictures have been found to be significantly stiffer than their surroundings [68, 71]. Thus, increased tissue strain may be assumed to be an accurate surrogate marker for intestinal fibrosis [71]. In US-SWE, instead, US shear waves are generated through an acoustic radiation impulse originating from the US probe and are applied onto a limited region of bowel wall [70, 72]. Its speed of propagation through the underlying tissue can be measured and speaks to its stiffness: the denser the material, the faster the propagation [68, 70, 72].

CEUS substantially improves upon standard US diagnostic potential by making use of an intravenously administered microbubble contrast agent with the aim of providing a more accurate depiction of the bowel wall microvasculature [65, 70, 72]. Indeed, tissue perfusion has been found to be negatively correlated to fibrosis and, thus, may serve as its surrogate index [68, 72]. Specific image analysis software programs are used to obtain an objectively quantitative measurement of the enhancement pattern (i.e., of the perfusion) [70, 72]. Nevertheless, studies have reported that CEUS is incapable of effectively detecting bowel wall fibrosis in the presence of inflammation [70].

#### CT

**Fig. 5:**
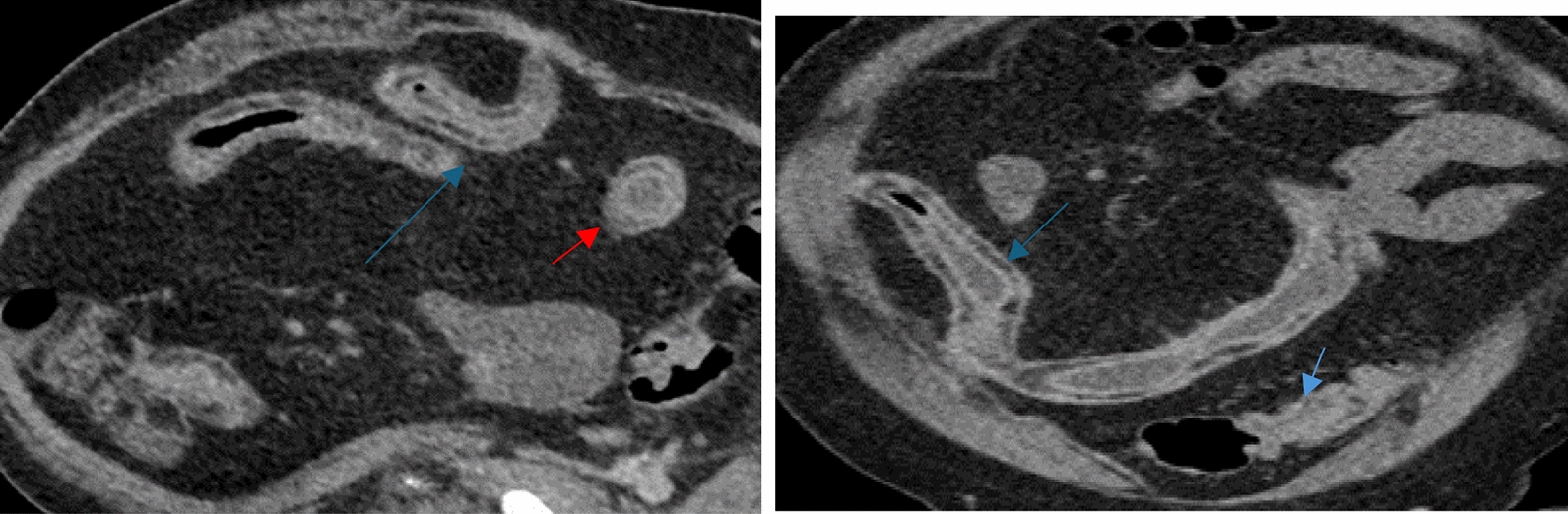
Axial and coronal CT images of the distal ileum showing extensive submucosal fat deposition (red arrrow) corresponding with sequela of chronic and severe inflammation in a 62-year-old patient with Crohn’s disease. Also, prominent regional fibrofatty proliferation separating the loops of bowel, “creeping fat” sign (blue arrow), typical of Crohn’s disease

CT and MRI are widely employed imaging techniques having excellent diagnostic accuracy (> 90%) for intestinal fibrosis distribution and severity [66, 68]. On CT, features such as mucosal enhancement, mesenteric hypervascularity, and mesenteric fat stranding are all suggestive of active CD related inflammation [66] (Fig. 5). This technology is widely available and offers 3D, multi-planar images with high spatial resolution and short acquisition time [65, 70]. Furthermore, it makes use of oral contrast agents to visualize the extent of bowel wall abnormalities and evaluate inflammatory activities [65, 70]. Recent development in the field of artificial intelligence has allowed for the realization of CT-based deep learning models which have proven to outperform human interpreters with increased accuracy and objectivity [73]. This technology’s main limitation, however, is that of exposure to ionizing radiation [65, 68]. Axial and coronal CT images of the distal ileum are provided in Fig. 5. In particular, they showcase a prominent regional fibrofatty proliferation separating the loops of the bowel known as "creeping fat" sign, typical of severe inflammation.

#### MRI

**Fig. 6:**
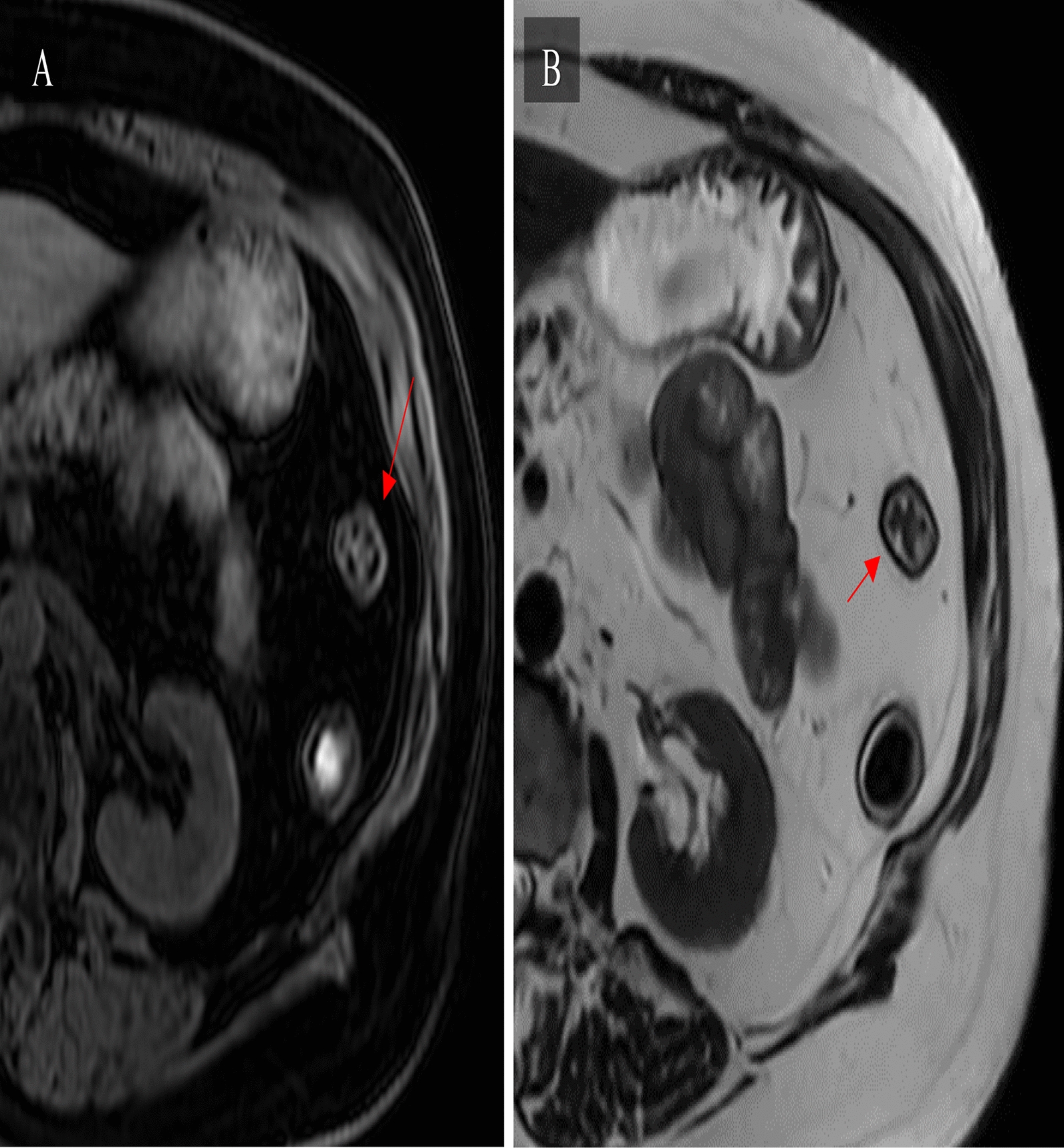
Axial T1 (**A**) and T2 weighted MRI (**B**) images highlighting submucosal fat deposition as well as thickened walls. See dark fibrotic wall on T2 (red arrow)

CE-MR has comparable sensitivity to that of CT with the added benefit of having superior soft tissue contrast capabilities and being radiation-free [65, 66, 68]. For this reason, it should be used preferentially in patients who are young, pregnant, or who are likely to need serial examination [66]. Similarly, to CT, CE-MR is performed after administration of oral contrast agents and allows for transmural observation of the bowel from various perspectives [65, 73] (Fig. 6). This technology is reported to be able to differentiate severe from mild to moderate fibrosis [69]. However, its ability to differentiate among none, mild, and moderate fibrosis is poor [69]. Further, it is a costly and more time-consuming alternative that is not as widely available [68]. Axial T1 and T2 weighted MRI images highlighting submucosal fat deposition as well as dark thickened fibrotic walls are shown in Fig. 6.

Diffusion-weighted imaging (DWI) capitalizes on the fact that the random motion of water molecules in the body is dependent on the cellular density of the tissue they are in [65, 73]. Indeed, excess collagen deposition, such as that found in fibrotic tissues, results in restricted extracellular water molecule motion [70]. The quantitative index with which this phenomenon is studied is the Apparent Diffusion Coefficient (ADC) [70, 73]. The ADC has been found to be significantly inversely related to the degree of inflammation and fibrosis, with high sensitivity (72%), high specificity (94%), and accuracy in agreement with that of contrast enhanced MR, proving its potential usefulness as a non-invasive technology contributing to intestinal fibrosis identification [65, 73]. Notably, DWI could be beneficial in patients for whom the use of MR contrast agents is contraindicated [65]. Even so, severe inflammatory background has been found to interfere with the accurate detection of fibrosis via ADC [70].

Magnetization transfer MRI (MT-MRI), a promising advancement in the field of MR imaging of CD related intestinal fibrosis, is a non-invasive technique that generates image contrast between protons in free water molecules and those within water molecules associated with large macromolecules, such as collagen [65, 70, 72, 73]. The resultant image enhancement can be quantified using the MT ratio, a measure of the transfer of nuclear spin polarization from one population of nuclei to another, which indirectly reflects the concentrations of macromolecules [65, 69]. Tissues containing high concentrations of collagen, such as fibrotic tissues, exhibit a higher mean MT ratio, making this technique of interest for bowel fibrosis detection, differentiation, and quantification [65, 69, 70, 72, 73]. Indeed, MT-MRI imaging outperforms Diffusion weighted MRI and contrast-enhanced imaging in distinguishing varying degrees of bowel fibrosis with or without coexisting inflammation [65, 69, 70]. This technique has also shown promise in distinguishing between mixed inflammatory fibrosis and pure inflammatory intestinal wall [69, 70].

At present, common MR techniques for evaluating intestinal wall perfusion of CD include dynamic contrast-enhanced MRI (DCE-MRI) and intravoxel incoherent motion (IVIM) [70]. DCE-MRI involves the serial acquisitions of T1-weighted images before, during, and after intravenous injection of gadolinium-based contrast agent [74]. Its perfusion parameters have been found to successful in assessing the characteristics of the bowel CD inflammation and in discriminating active and inactive CD [74]. Intravoxel incoherent motion-diffusion weighted Imaging (IVIM- DWI), instead, is a novel DWI technique which simultaneously measures both the random movement of water molecules in tissues and blood flow in capillary networks [74]. It has been reported to successfully detect significant differences in enhanced segments versus nonenhanced bowel segments as well as the degree of intestinal fibrosis [70, 74]. The advantage of IVIM over DCE-MRI is that it can produce image contrast without an IV enhancement [70]. It seems thatDCE-MRI and IVIM-DWI are both promising noninvasive ways to provide precise quantitative evaluation CD bowel inflammation [74]. In particular, IVIM-DWI without the need of contrast-agent injection to reflect the diffusion of water molecules and microcirculation perfusion in living tissues, has received special attention [70, 74].

#### Nuclear medicine

Fluorodeoxyglucose (FDG) PET localizes and quantifies FDG uptake in tissues of increased metabolic activity, such as areas of inflammation in CD [75]. The possibility to fuse functional data from PET and morphological data from CT or MR (PET-CT and PET-MR) has emerged as a promising imaging modality, having the potential to better assess the extent and location of disease than either sub-modality alone [70, 75]. PET/MR offers several advantages over PET/CT [75]. While PET/CT has been shown to be a useful modality for the identification of active bowel inflammation with results correlating well with the current gold standard and with an absolute reduction in false positive rates with respects to FDG-PET alone, its intrinsic need for sequential rather than concurrent acquisition may lead to motion artifacts and its use of ionizing radiation poses a substantial threat to CD patients, whose treatment plans often include serial examinations [69, 75]. Conversely, PET/MR’s synchronous image acquisition enables more accurate spatial and temporal matching of anatomical to functional data, and studies have shown it to present a 20%-73% reduction in radiation dose when compared to CT-MRI [75]. On top of having been reported to be significantly more accurate than either sub-modality alone in the detection of active inflammation (91% Vs 84% and 83%), PET-MR has also been found to be more accurate than PET-CT in detecting intestinal fibrosis [70, 75]. Further, PET-MR hybrid imaging has been reported to be useful in distinguishing fibrotic from inflammatory strictures, in accurately detecting extra-luminal disease, and to have superior soft tissue signal-to-noise ratio and contrast-to-noise ratio than CT-MRI [69, 75]. For all these reasons, this technology may potentially play a significant future role in the management of CD patients [75].

#### Future directions

Promising future techniques, benefits, and drawbacks of each imaging technique discussed above are summarized in Table 3. Among the proposed alternatives, the authors of this review believe MT-MRI to be the most promising. MT-MRI imaging outperforms competitors in distinguishing varying degrees of bowel fibrosis with or without coexisting inflammation [65, 69, 70]. This technique has also shown promise in distinguishing between mixed inflammatory fibrosis and pure inflammatory intestinal wall [69, 70]. It is a non-invasive technique that generates image contrast between protons in free water molecules and those within water molecules associated with large macromolecules, such as collagen, rather than requiring exogenous contrast administration [65, 70, 72, 73]. The resultant image enhancement can be quantified using the MT ratio, a proxy for fibrosis quantification [65, 69, 70, 72, 73].

**Table 3:** Intestinal fibrosis imaging—pros/cons with respects to the gold standard (endoscopy)

Intestinal fibrosis imaging		
US-SE/SWE	PROs	Readily available, low cost, lack of ionizing radiation
	CONs	Operator dependent, dependent on patient habitus
CEUS	PROs	Higher accuracy, quantitative
	CONs	Ineffective in case of active inflammation, use of contrast agent
CE-CT^a^	PROs	High accuracy, readily available, high spatial resolution, fast
	CONs	Use of contrast agents, use of ionizing radiation
CE-MRI	PROs	High accuracy, no ionizing radiation, higher soft tissue contrast, high sensitivity in early stages
	CONs	Use of contrast agents, low sensitivity in late stages, high cost, time consuming
IVIM/DWI-MRI	PROs	High sensitivity/specificity/accuracy, no use of contrast agents, quantitative
	CONs	Lower effectiveness in case of active inflammation, high cost, time consuming
MT-MRI^b^	PROs	Quantitative, no ionizing radiation, unaffected by active inflammation, no use of contrast agents
	CONs	High cost, time consuming
DCE-MRI	PROs	Distinguishes active/inactive inflammation, quantitative
	CONs	Use of contrast agent, high cost, time consuming
18-FDG-PET-CT	PROs	Reduced false positive rate
	CONs	Use of ionizing radiation, sequential (not concurrent) image acquisition, use of contrast agent
18-FDG-PET-MRI	PROs	Concurrent image acquisition, improved accuracy compared to PET and MRI alone, higher accuracy than 18-FDG-PET-CT, Higher signal-to-noise ratio, higher contrast-to-noise ratio, useful in distinguishing inflammatory/fibrotic strictures
	CONs	Use of contrast agent

^a^Gold standard

^b^Promising future techniques

## Conclusions

Fibrosis is the aberrant process of connective tissue deposition resulting from complications in tissue repair following repetitive injury, hypoxia, or ongoing infection [1]. It can affect any organ and is responsible for chronic and debilitating structural and functional impairment of the affected tissue [2, 3]. In fibrosis, pathological response to tissue damage determines an undue protraction of the healing process resulting in chronic inflammation, aberrant fibroblast proliferation, exaggerated collagen deposition, and a sequent imbalance in the alternation between scar formation and remodelling [3, 5]. While extensive research has already been carried out on the topic of aberrant wound healing and fibrogenesis, a thorough understanding of how this relationship reveals itself through imaging has yet to be established. Considering the far-reaching implications research furtherance in this field may have—starting from more early and accurate diagnosis—and with the aim of exploring and expanding upon all relevant knowledge, in this work we have attempted to outline the ways in which fibrosis shows up across abdominal organs and have listed the most relevant imaging technologies employed for its detection. A review of all pertinent literature has revealed US, CT, MR and PET to be among the most commonly adopted imaging technologies for the detection of fibrosis across all organs. Among the proposed alternatives, the authors of this review believe MRI to be the most promising imaging technique across all considered organs. Indeed, MRI has proven clear superiority when compared to competitors by virtue of elevated soft tissue contrast, lack of ionizing radiations, and its ability to successfully pair with elastography and DCE technology, among others. Furthermore, this imaging technique is widely available, allows for full-body scanning, and has been reported to produce fewer allergic reactions when compared to other contrast exploiting techniques (ex. C-ray and CT) (Table 4). Table 4 Authors’ opinion regarding the most promising radiology techniques to diagnose fibrosis in each organ Suspected affected organ Promising radiology techniques for diagnosis Pancreas MRCP and US (SE and SWE) Liver 3D-MRE Intestines MT-MRI.

**Table 4:** Authors’ opinion regarding the most promising radiology techniques to diagnose fibrosis in each organ

Suspected affected organ	Promising radiology techniques for diagnosis
Pancreas	MRCP and US (SE and SWE)
Liver	3D-MRE
Intestines	MT-MRI

## Disclosures

Mary Salvatore, MD, MBA- Consultant: Genentech, Boehringer Ingelheim. Grant funding: Boehringer Ingelheim, Genentech. Speaker: France Foundation, Peer View, Genentech, Boehringer Ingelheim. Research: Bioclinica, AbbVie, Lunglife AI.

## Supplementary Information

Additional file 1.

## Data Availability

Data sharing not applicable to this article as no datasets were generated or analyzed during the current study.

## Abbreviations

FDG: 18 F-Fluorodeoxyglucose
AE-IPF: Acute Exacerbation of IPF
ABUS: Automated Whole-Breast Us
BBD: Benign Breast Disease
BCT: Breast Computed Tomography
BI-RADS: Breast Imaging Reporting and Data System
BAL: Bronchoalveolar
CMR: Cardiac Magnetic Resonance
CEMDCT: Contrast Enhanced Multi-Detector CT
CEBCT: Contrast-Enhanced Breast CT
DBT: Digital Breast Tomosynthesis
TE: Echo Time
ECV: Extracellular volume
He: Helium
HRCT: High resolution computed tomography
IPF: Idiopathic pulmonary fibrosis
IGF-I: Insulin-like growth factor I
IGFBP-3: Insulin-like growth factor-binding protein 3
IL-8: Interleukin-8
LGE: Late gadolinium enhancement
M-CSF: Macrophage colony-stimulating factor
MRI: Magnetic resonance imaging
MMPS: Matrix metalloproteinases
MCP-1: Monocyte chemotactic protein-1
NK cells: Natural killer cells
PDGF: Platelet-derived growth factor
PET: Positron emission tomography
PAR: Protease activated receptors
Q-CT: Quantitative CT
ROS: Reactive oxygen species
RAAS: Renin–angiotensin–aldosterone system
STE: Speckle tracking echocardiography
TIMPS: Tissue inhibitors of metalloproteinases
TGF-Β1: Transforming growth factor Β1
US: Ultrasound
UTE: Ultrashort echo time
Xe: Xenon
ZTE: Zero echo time
