# Supplementary material for: Radiology of fibrosis part II: abdominal organs
Source: J Transl Med. 2024 Jul 2;22:610. doi: 10.1186/s12967-024-05346-w (PMC11218138; doi:10.1186/s12967-024-05346-w)
Supplement: Supplementary file 1 — Additional file 1. [file 12967_2024_5346_MOESM1_ESM.docx]

| **Term** | **Definition** |
| --- | --- |
| **Accuracy** | The degree to which a measured or estimated value closely matches the true or target value |
| **False Negative** | Occurs when a test result incorrectly indicates the absence of a condition or characteristic that is actually present. |
| **False Positives** | Occurs when a test result incorrectly indicates the presence of a condition or characteristic |
| **Positive Predictive Value** | The proportion of true positive results among all positive test results, indicating the probability that a positive test result accurately indicates the presence of the condition or disease being tested for |
| **Prevalence** | The proportion of individuals in a population who have a particular disease or condition at a specific point in time or over a specified period |
| **Sensitivity = True Positive Rate** | The ability of a diagnostic test to correctly identify individuals with the condition of interest, indicating the proportion of true positives among all individuals who truly have the condition |
| **Specificity** | The proportion of true negative results among all individuals without the condition being tested for |
| **Survival Advantage** | Quantitative measures that assess the extent to which a particular treatment or intervention improves survival outcomes compared to an alternative treatment or control group in medical research or clinical trials |

**Glossary 1:** Glossary of employed statistical terms

| **Term** | **Definition** |
| --- | --- |
| **18-FDG-PET-CT** | Medical imaging scans that combine PET with CT using the radiotracer FDG to visualize metabolic activity and anatomical structures in the body simultaneously |
| **2D Echocardiograph** | Non-invasive medical imaging technique that uses US waves to generate real-time, two-dimensional images of the heart's structure and function |
| **ABUS** | A technology that uses automated scanning to generate three-dimensional US images of the breast for supplemental screening in females with dense breast tissue |
| **CE enhanced MRI** | MRI technique that utilizes a contrast agent to enhance the visualization of blood vessels, tissues, or specific pathologies, providing improved diagnostic information |
| **CE enhanced CT** | CT technique that utilizes a contrast agent to enhance the visualization of blood vessels, tissues, or specific pathologies, providing improved diagnostic information |
| **CMR** | Cardiac-specific MRI technique |
| **CT** | Medical imaging technique that uses X-ray technology to generate detailed cross-sectional images of the body's internal structures |
| **DBT** | Imaging technique that produces three-dimensional images of the breast by capturing multiple X-ray images from different angles, aiding in the detection and diagnosis of breast cancer |
| **Digital Mammography** | Medical imaging technique that uses digital detectors to capture and produce high-resolution X-ray images of the breast for the detection and diagnosis of breast cancer |
| **Feature-Tracking CMR** | CMR technique that enables quantitative assessment of myocardial deformation by tracking tissue-specific features throughout the cardiac cycle |
| **Gradient Sequence MRI** | An MRI pulse sequence that utilizes gradients to encode spatial information and generates images by exploiting differences in the magnetic properties of tissues |
| **HRCT** | A specialized imaging technique that provides detailed, high-resolution images of the lungs and other structures with thinner slices than conventional CT scans |
| **LGE CMR** | Imaging technique used to visualize myocardial scar tissue by exploiting the differential uptake and washout of gadolinium-based contrast agents |
| **Mammoscintigraphy** | A nuclear medicine imaging technique that involves the injection of a radioactive tracer into the breast tissue to detect and evaluate abnormalities |
| **MRI** | A non-invasive medical imaging technique that uses strong magnetic fields and radio waves to generate detailed images of the internal structures of the body |
| **PET** | M edical imaging technique that uses radioactive tracers to detect metabolic activity and create detailed three-dimensional images of internal organs and tissues |
| **Quantitative CT** | The application of CT imaging techniques to quantitatively measure tissue properties such as density, volume, and perfusion for diagnostic and research purposes |
| **Real-Time 2D Colour Doppler Myocardial Imaging** | Cardiac imaging technique that provides live, two-dimensional visualization of blood flow within the heart, utilizing color coding to depict the direction and velocity of blood flow in real-time |
| **SPECT Gamma Camera** | Medical imaging device that detects gamma rays emitted from a radiotracer within the body to produce three-dimensional images |
| **Tissue Doppler Echocardiography** | A diagnostic imaging technique that uses Doppler US to measure the velocity and direction of myocardial tissue movement within the heart, providing valuable information about cardiac function and assessing myocardial mechanics |
| **US** | A medical imaging technique that uses high-frequency sound waves to produce real-time images of internal body structures |
| **UTE** | A technique in medical imaging that is designed to capture images with extremely short echo times, allowing for visualization of tissues with very short T2 relaxation times, such as cortical bone and tendons |
| **X-Rays** | A form of electromagnetic radiation used in medical imaging to produce images of the internal structures of the body by passing through tissues and creating contrast based on tissue density |
| **STE** | A non-invasive imaging technique that analyses myocardial motion by tracking the movement of natural acoustic markers (speckles) within ultrasound images of the heart, providing quantitative assessment of cardiac function |

**Glossary 2:** Glossary of employed radiological terms
